# Time to Confirmed Completion of Bowel Preparation as a Preprocedural Indicator of Colonoscope Insertion Difficulty: A Prospective Observational Study

**DOI:** 10.1002/deo2.70375

**Published:** 2026-07-07

**Authors:** Kazuki Horiuchi, Yusuke Ishibashi, Hiroki Ueda

**Affiliations:** ^1^ Department of Internal Medicine Japan Self‐Defense Force Iruma Hospital Saitama Japan; ^2^ Department of Internal Medicine National Defense Medical College Saitama Japan; ^3^ Department of Surgery Japan Self‐Defense Force Iruma Hospital Saitama Japan

**Keywords:** bowel preparation, cecal intubation time, colonoscopy, insertion difficulty, loop formation

## Abstract

**Background and Aims:**

Runway time, defined as the interval between completion of polyethylene glycol ingestion and the start of colonoscopy, has been studied as a determinant of bowel cleansing quality. However, whether the bowel preparation course itself is associated with colonoscope insertion difficulty remains unclear. We aimed to examine the associations of bowel preparation course indicators with cecal intubation time (CIT) and loop formation.

**Methods:**

In this single‐center prospective observational study, we enrolled consecutive patients undergoing unsedated colonoscopy and prospectively recorded four bowel preparation course indicators: time to first bowel movement, time to confirmed completion of bowel preparation, total number of bowel movements, and estimated stool volume. Multivariable linear regression analysis was performed using log‐transformed CIT as the dependent variable and adjusting for age, sex, body mass index (BMI), and endoscopist. Associations with loop formation were examined using multivariable logistic regression analysis adjusted for age, BMI, and endoscopist.

**Results:**

A total of 185 patients were included. Among the four indicators, only the time to confirmed completion of bowel preparation was associated with insertion difficulty. In multivariable linear regression analysis, each 30‐min increase in time to confirmed completion of bowel preparation was associated with longer CIT (exp(β), 1.139; 95% confidence interval [CI], 1.065–1.219). In multivariable logistic regression analysis, it was also associated with loop formation (odds ratio, 1.624; 95% CI, 1.129–2.360, per 30‐min increase).

**Conclusions:**

Time to confirmed completion of bowel preparation may serve as a simple preprocedural indicator of colonoscope insertion difficulty.

**Trial Registration:**

N/A.

## 1 Introduction

Cecal intubation time (CIT) is an established indicator of the technical difficulty of colonoscope insertion. Factors reportedly associated with difficult insertion include older age [1, 2, 3, 4, 5, 6, 7], female sex [1, 2, 3, 4, 5, 6, 7, 8], low body mass index (BMI) [1, 5, 7, 8, 9], a history of abdominal or pelvic surgery [2, 3, 9], constipation [7, 9], and inadequate bowel preparation [1, 4, 5]. Loop formation is another important mechanical factor contributing to insertion difficulty and has also been reported to be associated with older age [10] and female sex [10, 11, 12]. The mechanisms underlying difficult colonoscope insertion are thought to include anatomical factors intrinsic to the bowel itself, such as elongation of the rectosigmoid colon or total colon length, as well as increased mobility or redundancy of the transverse colon [13, 14]. In addition, extrinsic supportive factors, such as paucity of visceral fat, may also influence insertion difficulty [15]. These factors may contribute to prolonged CIT, particularly through loop formation in the sigmoid and transverse colon.

Polyethylene glycol (PEG)‐based regimens are widely used as standard bowel preparation methods for colonoscopy. Guidelines from the European Society of Gastrointestinal Endoscopy recommend starting the final dose of bowel preparation within 5 h of the procedure and completing it at least 2 h before colonoscopy [16]. The interval between completion of PEG ingestion and the start of colonoscopy, often referred to as runway time, has been recognized as an important determinant of bowel preparation quality. A meta‐analysis by Gao et al. demonstrated that a shorter runway time was significantly associated with better bowel cleansing quality [17]. However, runway time may not adequately reflect the patient's actual bowel movement course. The temporal pattern of bowel movements after the initiation of PEG ingestion may reflect interindividual differences in bowel responsiveness and intestinal transit characteristics. Accordingly, bowel preparation course indicators that capture the patient's actual bowel movement trajectory may serve as practical preprocedural indicators of difficult colonoscope insertion, but their clinical significance has not been adequately investigated.

To address this knowledge gap, the present study evaluated four bowel preparation course indicators reflecting the patient's actual bowel movement trajectory: time to first bowel movement, time to confirmed completion of bowel preparation, total number of bowel movements, and estimated stool volume. The aim of this study was to examine the associations of these four indicators with CIT and loop formation. We hypothesized that these bowel preparation course indicators would be associated with the technical difficulty of colonoscope insertion beyond established patient‐related factors.

## 2 Methods

### 2.1 Study Design and Population

This was a single‐center prospective observational study of consecutive patients who underwent colonoscopy between March 2024 and July 2025. At our institution, the standard bowel preparation regimen for morning colonoscopy consists of 24 mg of sennoside at bedtime on the day before colonoscopy, followed by ingestion of 2 L of PEG at the hospital on the morning of the procedure. In this study, the course of bowel preparation under routine clinical practice was prospectively monitored and recorded by medical staff.

Patients were excluded if they had a history of abdominal surgery, except for laparoscopic appendectomy or cholecystectomy; had confirmed or suspected enteritis, including inflammatory bowel disease; requested sedation; or underwent nonstandard bowel preparation or had insufficient data for evaluation of the bowel preparation course. Age, sex, and BMI at the time of colonoscopy were collected as covariates.

### 2.2 Bowel Preparation Course Indicators

The four bowel preparation course indicators were defined as follows: (1) time to first bowel movement, defined as the interval from the start of PEG ingestion to the first bowel movement (min); (2) time to confirmed completion of bowel preparation, defined as the interval from the start of PEG ingestion to the passage of nearly clear stools and subsequent confirmation of bowel preparation completion by medical staff (min); (3) total number of bowel movements, defined as the cumulative number of bowel movements until bowel preparation completion was confirmed; (4) estimated stool volume, defined as the estimated stool output (g), calculated from the change in body weight before and after PEG ingestion and the volume of PEG ingested.

### 2.3 Colonoscopy

At our institution, colonoscopy was routinely performed using the EC‐L600ZP colonoscope (FUJIFILM Corporation, Tokyo, Japan) fitted with a distal attachment cap. In routine practice, an antispasmodic agent (hyoscine butylbromide or glucagon) was administered immediately before the examination. Colonoscope insertion was routinely performed using the water immersion method [18, 19] in combination with the axis‐maintaining shortening technique [20]. Colonoscopy was performed by three endoscopists with 15, 10, and 8 years of experience in colonoscopy, respectively. All three endoscopists had performed more than 1000 colonoscopies using the water immersion method and the axis‐maintaining shortening technique.

CIT, measured in seconds, was defined as the interval from insertion of the colonoscope through the anus to acquisition of a photograph of the appendiceal orifice after cecal intubation. The presence or absence of loop formation was recorded on the basis of the endoscopist's subjective assessment. With reference to previous reports [19], loop formation was defined as a condition during passage through the sigmoid colon in which the tip of the colonoscope failed to advance along the course of the bowel in response to scope manipulation and was accompanied by obvious bowing of the shaft. Cases in which only a minor loop was resolved by short push‐pull maneuvers were not considered to represent loop formation. In contrast, cases in which the scope was advanced under loop formation to the sigmoid‐descending junction or the splenic flexure, and subsequent withdrawal was required for loop reduction, were classified as having loop formation. Because previous reports have shown that most pain episodes during colonoscopy occur in the sigmoid colon and are associated with loop formation [12], loop formation was evaluated only in the sigmoid colon.

### 2.4 Statistical Analysis

The associations between the four bowel preparation course indicators and CIT were assessed using Spearman's rank correlation coefficients. Because CIT showed a right‐skewed distribution, multivariable linear regression analysis was performed using the natural logarithm of CIT as the dependent variable. The time to confirmed completion of bowel preparation was entered as an explanatory variable per 30‐min increment. The model was adjusted for BMI, age, sex, and endoscopist (endoscopists A and B, with C as the reference). Effect estimates were presented as exp(β).

To examine the association between time to confirmed completion of bowel preparation and loop formation, multivariable logistic regression analysis was performed. Because the study population was markedly imbalanced toward male patients, sex was not included in the primary model, and its influence was examined in sensitivity analyses. In the primary analysis, time to confirmed completion of bowel preparation was entered as the explanatory variable per 30‐min increment, and the model was adjusted for BMI, age, and endoscopist. Sensitivity analyses included an additional model further adjusted for sex, a model restricted to male patients, and a Firth penalized logistic regression model.

To assess the effect modification by the endoscopist, an additional model including an interaction term between time to confirmed completion of bowel preparation and the endoscopist was fitted. For analyses of CIT, models with and without the interaction term were compared using an F‐test based on analysis of variance. For analyses of loop formation, models with and without the interaction term were compared using the likelihood ratio test.

All tests were two‐sided, and *p*‐values < 0.05 were considered statistically significant. Ninety‐five percent confidence intervals (CIs) were calculated. All statistical analyses were performed using R version 4.5.2 (R Foundation for Statistical Computing, Vienna, Austria).

## 3 Results

### 3.1 Patient Characteristics

A total of 263 patients underwent colonoscopy during the study period. Of these, 30 were excluded because of a history of abdominal surgery, 11 because of confirmed or suspected enteritis, 27 because they requested sedation, 6 because bowel preparation deviated from the institutional standard regimen (all due to omission of bedtime sennoside on the day before colonoscopy), and 4 because they declined to participate. Ultimately, 185 patients were included in the analysis.

The median age was 49 years, and 178 patients (96%) were male (Table 1). The median BMI was 24.4 kg/m^2^. The median time to first bowel movement was 48 min, and the median time to confirmed completion of bowel preparation was 118 min. The median total number of bowel movements was 6, and the median estimated stool volume was 1,650 g. The median CIT was 243 s, and loop formation was observed in 29% of patients. Table S1 presents patient characteristics according to quartiles of time to confirmed completion of bowel preparation.

**TABLE 1:** Baseline characteristics of the study population.

	Overall *n* = 185**^a^**
Age (years)	49 [43, 54]
Sex, male	178 (96%)
BMI (kg/m^2^)	24.4 [22.8, 26.3]
Time to confirmed completion of bowel preparation (min)	118 [106, 143]
Time to first bowel movement (min)	48 [37, 60]
Total number of bowel movements	6 [5, 7]
Estimated stool volume (g)	1650 [1350, 2100]
CIT (s)	243 [189, 355]
Loop formation	53 (29%)
Endoscopist	
A	73 (39%)
B	48 (26%)
C	64 (35%)

Abbreviations: BMI, body mass index; CIT, cecal intubation time.

^a^ Data are presented as median [IQR] or *n* (%).

### 3.2 Four Bowel Preparation Course Indicators

The correlations between the four bowel preparation course indicators and CIT are shown in Supplementary Figure S1. Time to confirmed completion of bowel preparation was positively correlated with CIT (*ρ* = 0.33, *p* < 0.001), whereas the other three bowel preparation course indicators were not significantly correlated with CIT.

### 3.3 Time to Confirmed Completion of Bowel Preparation and CIT

**FIGURE 1:**
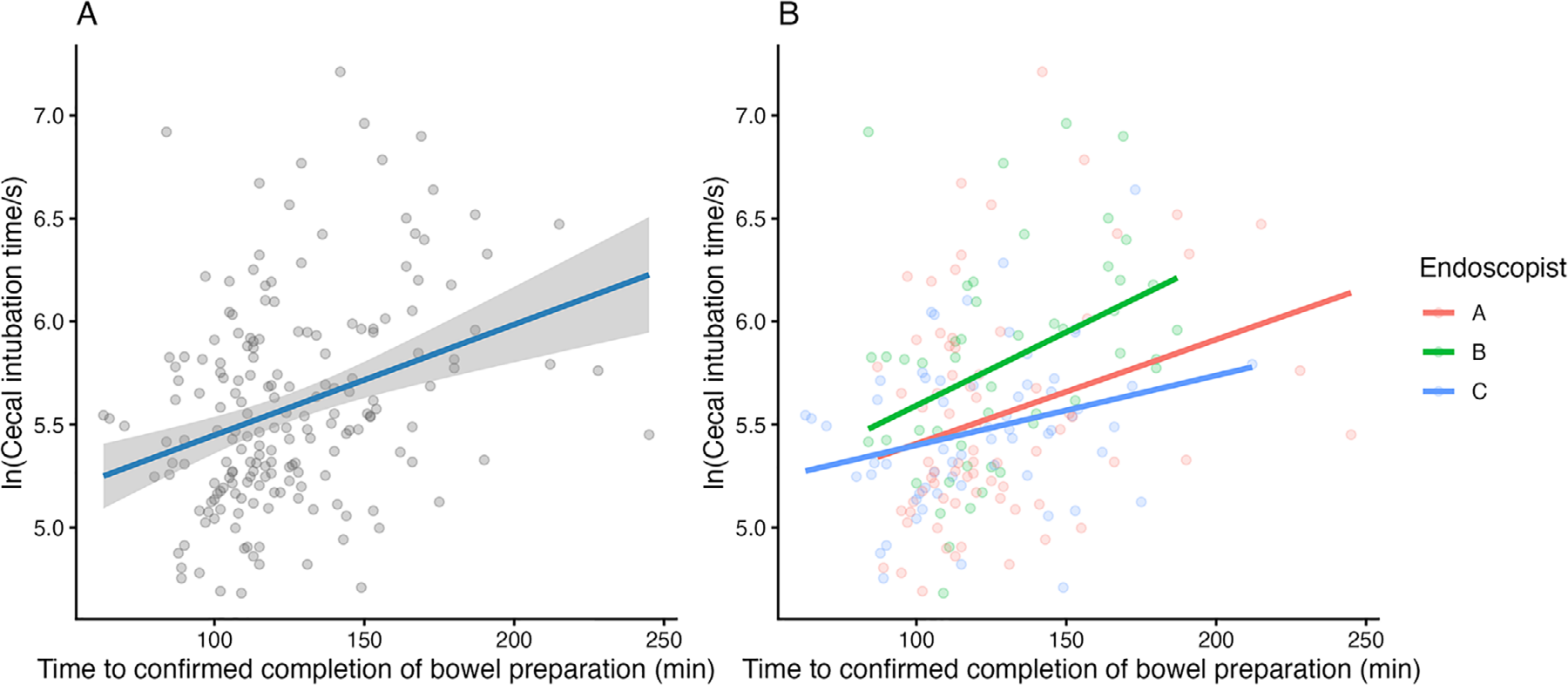
Relationship between time to confirmed completion of bowel preparation and ln(Cecal intubation time). (A) Overall scatter plot with fitted linear regression line and 95% confidence band. (B) Endoscopist‐specific scatter plots with fitted linear regression lines.

The relationship between time to confirmed completion of bowel preparation and log‐transformed CIT is shown in Figure 1. Overall, a longer time to confirmed completion of bowel preparation was associated with longer CIT (Figure 1A). A similar trend was observed in the endoscopist‐specific analyses, with generally comparable slopes of the regression lines across endoscopists (Figure 1B).

**FIGURE 2:**
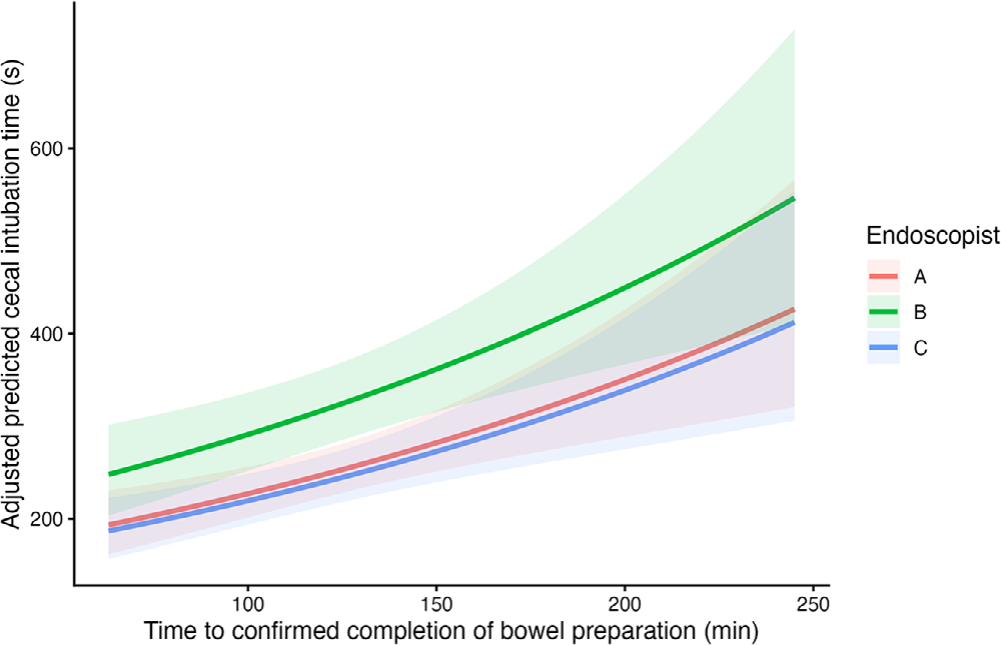
Adjusted predicted cecal intubation time by time to confirmed completion of bowel preparation. Curves were derived from a multivariable linear regression model for ln(Cecal intubation time) and back‐transformed to seconds. Predictions were calculated with body mass index and age fixed at their median values. Endoscopist‐specific curves are shown. Shaded areas indicate 95% confidence intervals for the mean predicted values. No significant interaction between time to confirmed completion of bowel preparation and endoscopist was observed (analysis of variance [ANOVA], *F* = 0.84; *p* = 0.432).

Figure 2 shows the covariate‐adjusted prediction curves for time to confirmed completion of bowel preparation and CIT, and the corresponding results of the multivariable linear regression analysis are presented in Table 2. The interaction between time to confirmed completion of bowel preparation and endoscopist was not statistically significant (analysis of variance, *F* = 0.84; *p* = 0.432), indicating that the association between time to confirmed completion of bowel preparation and CIT was generally consistent across endoscopists.

**TABLE 2:** Multivariable linear regression analysis of ln(Cecal intubation time).

Variable	β (ln scale)	SE	*t*	*p*	Ratio exp(β) (95% CI)
Time to confirmed completion of bowel preparation (per 30 min)	0.130	0.034	3.812	<0.001	1.139 (1.065–1.219)
BMI (per 1 kg/m^2^)	−0.023	0.012	−1.981	0.049	0.977 (0.954–1.000)
Age (per 1 year)	0.007	0.005	1.507	0.134	1.007 (0.998–1.017)
Male (vs. Female)	−0.137	0.180	−0.763	0.447	0.872 (0.611–1.243)
Endoscopist A (vs. C)	0.034	0.078	0.435	0.664	1.035 (0.886–1.208)
Endoscopist B (vs. C)	0.282	0.088	3.222	0.002	1.326 (1.116–1.577)

Abbreviations: BMI, body mass index; CI, confidence interval; SE, standard error.

After adjustment for covariates, time to confirmed completion of bowel preparation remained independently associated with prolonged CIT, with an exp(β) of 1.139 (95% CI, 1.065–1.219; *p* < 0.001) for each 30‐min increase. BMI was marginally associated with shorter CIT, with an exp(β) of 0.977 (95% CI, 0.954–1.000; *p* = 0.049), whereas age and sex were not significantly associated with CIT. In addition, endoscopist B had a significantly longer CIT than endoscopist C.

### 3.4 Time to Confirmed Completion of Bowel Preparation and Loop Formation

**FIGURE 3:**
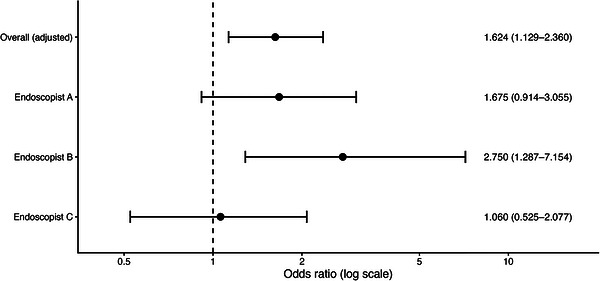
Association between time to confirmed completion of bowel preparation and loop formation. Points represent odds ratios, and horizontal lines indicate 95% confidence intervals, per 30‐min increase in time to confirmed completion of bowel preparation. The overall estimate was obtained from a multivariable logistic regression model adjusted for body mass index (BMI), age, and endoscopist. Endoscopist‐specific estimates were derived from models stratified by endoscopist and adjusted for BMI and age. The interaction between time to confirmed completion of bowel preparation and endoscopist was not statistically significant (likelihood ratio test, *p* = 0.066).

Figure 3 shows the odds ratios for time to confirmed completion of bowel preparation in the overall and endoscopist‐specific analyses. In the overall analysis, time to confirmed completion of bowel preparation was significantly associated with loop formation, with an odds ratio of 1.624 (95% CI, 1.129–2.360). In the endoscopist‐specific analyses, the association was strongest for endoscopist B, with an odds ratio of 2.750 (95% CI, 1.287–7.154), whereas no statistically significant association was observed for endoscopist A (odds ratio, 1.675; 95% CI, 0.914–3.055) or endoscopist C (odds ratio, 1.060; 95% CI, 0.525–2.077).

The odds ratios for the covariates are shown in Table 3. BMI and age were not significantly associated with loop formation. In contrast, endoscopist B had significantly higher odds of loop formation than endoscopist C (odds ratio, 6.463; 95% CI, 2.791–15.786). The likelihood ratio test for the interaction between time to confirmed completion of bowel preparation and endoscopist yielded *χ* ^2^ = 5.43 (*p* = 0.066), which did not indicate a statistically significant interaction.

**TABLE 3:** Multivariable logistic regression analysis of loop formation.

Variable	OR (95% CI)	*p*
Time to confirmed completion of bowel preparation (per 30 min)	1.624 (1.129–2.360)	0.009
BMI (per 1 kg/m^2^)	1.065 (0.939–1.209)	0.326
Age (per 1 year)	0.988 (0.937–1.043)	0.654
Endoscopist A (vs. C)	0.403 (0.145–1.047)	0.068
Endoscopist B (vs. C)	6.463 (2.791–15.786)	<0.001

Abbreviations: BMI, body mass index; CI, confidence interval; OR, odds ratio.

### 3.5 Sensitivity Analyses for Sex in Relation to Loop Formation

The odds ratio for time to confirmed completion of bowel preparation was 1.67 (95% CI, 1.15–2.45) in the model additionally adjusted for sex and 1.78 (95% CI, 1.22–2.65) in the analysis restricted to male patients (Table S2). In the Firth penalized logistic regression analysis, the corresponding odds ratio was 1.60 (95% CI, 1.12–2.29).

## 4 Discussion

We used four bowel preparation course indicators reflecting the actual bowel movement course and examined their associations with insertion difficulty. Among these indicators, time to confirmed completion of bowel preparation was significantly associated with CIT in the multivariable linear regression analysis adjusted for BMI, age, sex, and endoscopist. Specifically, each 30‐min increase in time to confirmed completion of bowel preparation was associated with an approximately 14% prolongation of CIT. The slopes of the endoscopist‐specific regression lines and the covariate‐adjusted prediction curves were broadly similar, and no statistically significant interaction was observed between time to confirmed completion of bowel preparation and endoscopist, suggesting that the association between time to confirmed completion of bowel preparation and CIT was generally consistent across endoscopists. In the multivariable logistic regression analysis adjusted for BMI, age, and endoscopist, time to confirmed completion of bowel preparation was significantly associated with loop formation overall, with an odds ratio of 1.62 for each 30‐min increase. Although the estimated associations appeared to vary across endoscopists, the likelihood ratio test for interaction between time to confirmed completion of bowel preparation and endoscopist was not statistically significant (*χ* ^2^ = 5.43, *p* = 0.066). Thus, although possible effect modification by the endoscopist was suggested for loop formation, no statistically significant interaction was demonstrated.

The bowel preparation course indicators used in the present study differ from the runway time commonly used in previous studies [17]. Prolongation of runway time is thought to impair bowel cleansing quality through reaccumulation of intestinal fluid and small bowel contents over time [21]. In this sense, runway time may be regarded as an indicator of recontamination or reaccumulation after completion of bowel preparation. In contrast, the bowel preparation course evaluated in our study reflects the process from the start of PEG ingestion to confirmed completion of bowel preparation, and may therefore capture interindividual differences in bowel responsiveness and intestinal transit. Indeed, previous studies have shown that a time to first bowel movement of 90 min or longer [22] and five or fewer bowel movements [23] are associated with inadequate bowel preparation. The present study focused on the association between the bowel preparation course itself and insertion difficulty. To our knowledge, few studies have specifically examined this association.

A major strength of this study is that the bowel preparation course was prospectively recorded, allowing the association between bowel preparation course indicators and insertion difficulty to be evaluated within the same cohort. In addition, procedure‐related variability may have been limited because colonoscopy was performed by only three experienced endoscopists within a relatively uniform clinical setting, including routine antispasmodic use, unsedated colonoscopy, use of the same colonoscope, and the same insertion technique. Furthermore, the time to confirmed completion of bowel preparation is noninvasive, easy to obtain, and readily applicable in clinical practice. The time to confirmed completion of bowel preparation can be confirmed by medical staff and can also be recorded by the patient when appropriate instructions are provided.

This study has several limitations. First, the number of elderly patients was extremely small, with only one patient aged 65 years or older. Because increasing age is known to be associated with greater colonic length [24, 25], it remains unclear whether the same findings would be observed in an older population. Second, the study population was overwhelmingly male. Although sensitivity analyses incorporating sex yielded similar estimates, the number of female patients was insufficient for a reliable female‐only analysis. Third, loop formation was assessed on the basis of the endoscopist's subjective judgment, although interobserver variability may have been limited to some extent because colonoscopy was performed in a relatively uniform procedural setting. Fourth, evaluation of loop formation was limited to the sigmoid colon, and loop formation at other sites, including the transverse colon, was not assessed. However, previous reports have identified sigmoid loop formation as the site most strongly associated with pain during colonoscopy [12], and the present study therefore focused on the form of loop formation considered to be of greatest clinical relevance. Fifth, unmeasured confounding may have been present, including constipation tendency, the presence of colonic diverticula, and dietary intake during the several days preceding colonoscopy. Finally, this was a single‐center study conducted in a relatively homogeneous clinical setting, involving unsedated colonoscopy performed by experienced endoscopists under broadly similar procedural conditions; therefore, caution is warranted in generalizing these findings to other institutions or different examination settings.

## 5 Conclusion

Prolonged time to confirmed completion of bowel preparation was significantly associated with longer CIT, with no clear evidence of effect modification by endoscopist. Prolonged time to confirmed completion of bowel preparation was also significantly associated with a greater likelihood of loop formation, although possible effect modification by the endoscopist was suggested. These findings suggest that time to confirmed completion of bowel preparation may serve as a simple preprocedural indicator of insertion difficulty.

## Funding

The authors have nothing to report.

## Ethics Statement

The study protocol was approved by the ethics committee of our hospital (approval number 6‐2‐1).

## Consent

Written informed consent was obtained from all participants.

## Conflicts of Interest

The authors declare no conflicts of interest.

## Supporting information




**Figure S1**: Correlation between bowel preparation course indicators and cecal intubation time.
Points represent Spearman's correlation coefficients (ρ), and horizontal lines indicate 95% confidence intervals, for the association between each bowel preparation course indicator and cecal intubation time. *p*‐Values are shown on the right.

**Table S1**: Baseline characteristics by quartiles of time to confirmed completion of bowel preparation.
Data are presented as median [IQR] or *n* (%).

**Table S2**: Sensitivity analysis for sex in relation to loop formation.
Sensitivity analyses for sex in the association between time to confirmed completion of bowel preparation and loop formation. Odds ratios are shown per 30‐min increase in time to confirmed completion of bowel preparation.

## Data Availability

The data underlying this study are not publicly available but are available from the corresponding author upon reasonable request.

## References

[1] V. Jaruvongvanich , T. Sempokuya , P. Laoveeravat , and P. Ungprasert , “Risk Factors Associated With Longer Cecal Intubation Time: A Systematic Review and Meta‐Analysis,” International Journal of Colorectal Disease 33, no. 4 (2018): 359–365, 10.1007/s00384-018-3014-x. 29520457

[2] H. A. Shah , L. F. Paszat , R. Saskin , T. A. Stukel , and L. Rabeneck , “Factors Associated With Incomplete Colonoscopy: A Population‐Based Study,” Gastroenterology 132, no. 7 (2007): 2297–2303, 10.1053/j.gastro.2007.03.032. 17570204

[3] S. Koido , T. Ohkusa , K. Nakae , et al., “Factors Associated With Incomplete Colonoscopy at a Japanese Academic Hospital,” World Journal of Gastroenterology 20, no. 22 (2014): 6961–6967, 10.3748/wjg.v20.i22.6961. 24944489 PMC4051938

[4] Y.‐H. Hsieh , C.‐S. Kuo , K.‐C. Tseng , and H.‐J. Lin , “Factors That Predict Cecal Insertion Time During Sedated Colonoscopy: The Role of Waist Circumference,” Journal of Gastroenterology and Hepatology 23, no. 2 (2008): 215–217, 10.1111/j.1440-1746.2006.04818.x. 18289354

[5] P. Krishnan , A. A. Sofi , R. Dempsey , O. Alaradi , and A. Nawras , “Body Mass Index Predicts Cecal Insertion Time: The Higher, the Better,” Digestive Endoscopy 24, no. 6 (2012): 439–442, 10.1111/j.1443-1661.2012.01296.x. 23078436

[6] K. Kashiwagi , N. Inoue , T. Yoshida , et al., “The Impact of Visceral Adipose Tissue as Best Predictor for Difficult Colonoscopy and the Clinical Utility of a Long Small‐Caliber Scope as Rescue,” PLoS ONE 12, no. 12 (2017): e0189817, 10.1371/journal.pone.0189817. 29267320 PMC5739452

[7] J. C. Anderson , C. R. Messina , W. Cohn , et al., “Factors Predictive of Difficult Colonoscopy,” Gastrointestinal Endoscopy 54, no. 5 (2001): 558–562, 10.1067/mge.2001.118950. 11677470

[8] S. Y. Moon , B. C. Kim , D. K. Sohn , et al., “Predictors for Difficult Cecal Insertion in Colonoscopy: The Impact of Obesity Indices,” World Journal of Gastroenterology 23, no. 13 (2017): 2346–2354, 10.3748/wjg.v23.i13.2346. 28428714 PMC5385401

[9] B. Dinçer , S. Ömeroğlu , O. Güven , et al., “Factors Predict Prolonged Colonoscopy Before the Procedure: Prospective Registry Study,” Surgical Endoscopy 38, no. 10 (2024): 5704–5711, 10.1007/s00464-024-11075-4. 39138684

[10] O. Toyoshima , T. Nishizawa , S. Yoshida , et al., “Impact of Looping on Premalignant Polyp Detection During Colonoscopy,” World Journal of Gastrointestinal Endoscopy 14, no. 11 (2022): 694–703, 10.4253/wjge.v14.i11.694. 36438882 PMC9693685

[11] J. Lam , J. Wilkinson , C. Brassett , and J. Brown , “Difference in Real‐Time Magnetic Image Analysis of Colonic Looping Patterns Between Males and Females Undergoing Diagnostic Colonoscopy,” Endoscopy International Open 6, no. 5 (2018): E575–E581, 10.1055/a-0574-2478. 29756015 PMC5943688

[12] S. G. Shah , J. C. Brooker , C. Thapar , C. B. Williams , and B. P. Saunders , “Patient Pain During Colonoscopy: An Analysis Using Real‐Time Magnetic Endoscope Imaging,” Endoscopy 34, no. 6 (2002): 435–440, 10.1055/s-2002-31995. 12048623

[13] B. P. Saunders , S. Halligan , C. Jobling , et al., “Can Barium Enema Indicate When Colonoscopy Will be Difficult?” Clinical Radiology 50, no. 5 (1995): 318–321, 10.1016/S0009-9260(05)83424-5. 7743720

[14] B. P. Saunders , M. Fukumoto , S. Halligan , et al., “Why Is Colonoscopy More Difficult in Women?” Gastrointestinal Endoscopy 43, no. 2 (1996): 124–126, 10.1016/S0016-5107(06)80113-6. 8635705

[15] G. E. Chung , S. H. Lim , S. Y. Yang , et al., “Factors That Determine Prolonged Cecal Intubation Time During Colonoscopy: Impact of Visceral Adipose Tissue,” Scandinavian Journal of Gastroenterology 49, no. 10 (2014): 1261–1267, 10.3109/00365521.2014.950695. 25144912

[16] C. Hassan , J. East , F. Radaelli , et al., “Bowel Preparation for Colonoscopy: European Society of Gastrointestinal Endoscopy (ESGE) Guideline—Update 2019,” Endoscopy 51, no. 8 (2019): 775–794, 10.1055/a-0959-0505. 31295746

[17] Y. Gao and X.‐J. Lin , “Effect of Bowel Preparation to Colonoscopy Interval on Preparation Quality and Colonoscopy Outcomes: A Meta‐Analysis,” Turkish Journal of Gastroenterology 34, no. 1 (2023): 26–34, 10.5152/tjg.2022.22033. PMC9984965 36511605

[18] C. W. Leung , T. Kaltenbach , R. Soetikno , K. K. Wu , F. W. Leung , and S. Friedland , “Water Immersion Versus Standard Colonoscopy Insertion Technique: Randomized Trial Shows Promise for Minimal Sedation,” Endoscopy 42, no. 7 (2010): 557–563, 10.1055/s-0029-1244231. 20593332

[19] S. Asai , N. Fujimoto , K. Tanoue , et al., “Water Immersion Colonoscopy Facilitates Straight Passage of the Colonoscope Through the Sigmoid Colon Without Loop Formation: Randomized Controlled Trial,” Digestive Endoscopy 27, no. 3 (2015): 345–353, 10.1111/den.12406. 25413483

[20] S. Yamamoto , Y. Sano , N. Mottacki , and H. Neumann , “Axis‐Keeping Shortening Technique for Colonic Intubation,” VideoGIE 5, no. 12 (2020): 630–633, 10.1016/j.vgie.2020.08.005. 33015426 PMC7522744

[21] T. K. Kim , H. W. Kim , S. J. Kim , et al., “Importance of the Time Interval Between Bowel Preparation and Colonoscopy in Determining the Quality of Bowel Preparation for Full‐Dose Polyethylene Glycol Preparation,” Gut and Liver 8, no. 6 (2014): 625–631, 10.5009/gnl13228. 25368750 PMC4215448

[22] V. Papastergiou , S. Papasavvas , N. Mathou , et al., “A Delayed Onset of Bowel Activity After the Start of Conventional Polyethylene Glycol Predicts Inadequate Colon Cleansing Before Colonoscopy: A Prospective Observational Study,” United European Gastroenterology Journal 4, no. 2 (2016): 199–206, 10.1177/2050640615608572. 27087947 PMC4804377

[23] H. G. Kim , S. R. Jeon , M. Y. Kim , et al., “How to Predict Adequate Bowel Preparation Before Colonoscopy Using Conventional Polyethylene Glycol: Prospective Observational Study Based on Survey,” Digestive Endoscopy 27, no. 1 (2015): 87–94, 10.1111/den.12312. 24833415

[24] P. J. Pickhardt and V. Razdan , “CT Colonography for Longitudinal In‐Vivo Assessment of Colonic Lengthening in Middle‐Aged and Older Adults,” Abdominal Radiology 51, no. 7 (2026): 3308–3313, 10.1007/s00261-025-05334-8, (published online: December 19, 2025). 41417073 PMC13269295

[25] M. A. Khashab , P. J. Pickhardt , D. H. Kim , and D. K. Rex , “Colorectal Anatomy in Adults at Computed Tomography Colonography: Normal Distribution and the Effect of Age, Sex, and Body Mass Index,” Endoscopy 41, no. 8 (2009): 674–678, 10.1055/s-0029-1214899. 19670134

