# Supplementary figures and images for: Time to Confirmed Completion of Bowel Preparation as a Preprocedural Indicator of Colonoscope Insertion Difficulty: A Prospective Observational Study
Source: DEN Open. 2026 Jul 7;7(1):e70375. doi: 10.1002/deo2.70375 (PMC13339062; doi:10.1002/deo2.70375)

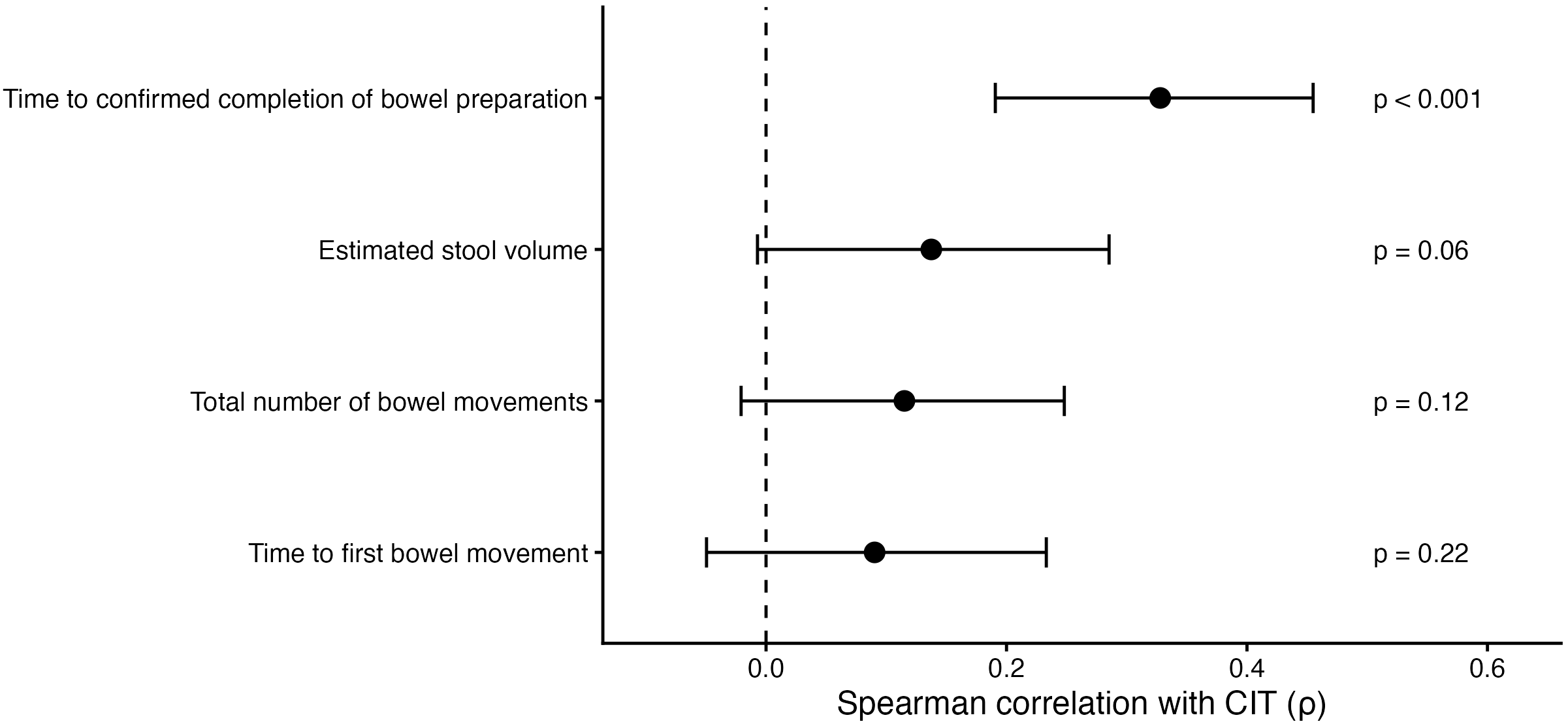

Supplement: Supplementary file 1 — Figure S1: Correlation between bowel preparation course indicators and cecal intubation time. Points represent Spearman's correlation coefficients (ρ), and horizontal lines indicate 95% confidence intervals, for the association between each bowel preparation course indicator and cecal intubation time. p‐Values are shown on the right. [file DEO2-7-e70375-s003.png]
