# Supplementary material for: Time to Confirmed Completion of Bowel Preparation as a Preprocedural Indicator of Colonoscope Insertion Difficulty: A Prospective Observational Study
Source: DEN Open. 2026 Jul 7;7(1):e70375. doi: 10.1002/deo2.70375 (PMC13339062; doi:10.1002/deo2.70375)
Supplement: Supplementary file 2 — Table S1: Baseline characteristics by quartiles of time to confirmed completion of bowel preparation. Data are presented as median [IQR] or n (%). [file DEO2-7-e70375-s001.docx]

|  | Q1 (fast)  *n* = 47^†^ | Q2   *n* = 46^†^ | Q3   *n* = 46^†^ | Q4 (slow)   *n* = 46^†^ |
| --- | --- | --- | --- | --- |
| Age (years) | 49 [43, 54] | 48 [42, 52] | 49 [44, 54] | 52 [43, 55] |
| Sex, male | 45 (96%) | 45 (98%) | 45 (98%) | 43 (93%) |
| BMI (kg/m^2^) | 25.3 [23.4, 26.4] | 24.9 [23.1, 27.0] | 24.4 [22.4, 26.6] | 23.6 [21.7, 25.2] |
| Time to confirmed completion of BP (min) | 97 [88, 102] | 112 [109, 115] | 128 [122, 134] | 164 [152, 175] |
| Time to first bowel movement (min) | 42 [33, 47] | 47 [33, 55] | 50 [45, 65] | 56 [44, 70] |
| Total number of bowel movements | 6 [5, 7] | 6 [5, 7] | 6 [5, 7] | 7 [6, 9] |
| Estimated stool volume (g) | 1,400 [1,130, 1,800] | 1,550 [1,380, 1,900] | 1,750 [1,550, 2,000] | 2,200 [1,500, 2,450] |
| CIT (s) | 225 [168, 315] | 208 [175, 339] | 244 [194, 312] | 325 [239, 493] |
| Loop formation | 10 (21%) | 11 (24%) | 11 (24%) | 21 (46%) |
| Endoscopist |  |  |  |  |
| A | 16 (34%) | 22 (48%) | 21 (46%) | 14 (30%) |
| B | 9 (19%) | 12 (26%) | 12 (26%) | 15 (33%) |
| C | 22 (47%) | 12 (26%) | 13 (28%) | 17 (37%) |

**Supplementary Table 1.** Baseline characteristics by quartiles of time to confirmed completion of bowel preparation

Abbreviations: BP, bowel preparation; BMI, body mass index; CIT, cecal intubation time.

^†^Data are presented as median [IQR] or n (%).
