# Supplementary material for: Time to Confirmed Completion of Bowel Preparation as a Preprocedural Indicator of Colonoscope Insertion Difficulty: A Prospective Observational Study
Source: DEN Open. 2026 Jul 7;7(1):e70375. doi: 10.1002/deo2.70375 (PMC13339062; doi:10.1002/deo2.70375)
Supplement: Supplementary file 3 — Table S2: Sensitivity analysis for sex in relation to loop formation. Sensitivity analyses for sex in the association between time to confirmed completion of bowel preparation and loop formation. Odds ratios are shown per 30‐min increase in time to confirmed completion of bowel preparation. [file DEO2-7-e70375-s002.docx]

**Supplementary Table 2.** Sensitivity analysis for sex in relation to loop formation

| Analysis | OR (95% CI) | p |
| --- | --- | --- |
| Primary: no sex | 1.62 (1.13–2.36) | 0.009 |
| Sensitivity: + sex | 1.67 (1.15–2.45) | 0.008 |
| Sensitivity: males only | 1.78 (1.22–2.65) | 0.003 |
| Sensitivity: Firth (no sex) | 1.60 (1.12–2.29) | 0.010 |

Abbreviations: OR, odds ratio; CI, confidence interval
